# Reinforcement learning of 2-joint virtual arm reaching in motor cortex simulation

**DOI:** 10.1186/1471-2202-13-S1-P90

**Published:** 2012-07-16

**Authors:** Samuel A Neymotin, George L Chadderdon, Cliff C Kerr, Joseph T Francis, William W Lytton

**Affiliations:** 1 SUNY Downstate Medical Center; 450 Clarkson Avenue; Brooklyn, NY 11203, USA; 2 School of Physics, University of Sydney, NSW 2006, Australia; 3 Kings County Hospital, Brooklyn, NY 11203, USA

## 

Few attempts have been made to model learning of sensory-motor control using spiking neural units. We trained a 2-degree-of-freedom virtual arm to reach for a target using a spiking-neuron model of motor cortex that maps proprioceptive representations of limb position to motor commands and undergoes learning based on reinforcement mechanisms suggested by the dopaminergic reward system. A 2-layer model of layer 5 motor cortex (M1) passed motor commands to the virtual arm and received proprioceptive position information from it. The reinforcement algorithm trained synapses of M1 using reward (punishment) signals based on visual perception of decreasing (increasing) distance of the virtual hand from the target. Output M1 units were partially driven by noise, creating stochastic movements that were shaped to achieve desired outcomes.

The virtual arm consisted of a shoulder joint, upper arm, elbow joint, and forearm. The upper- and forearm were each controlled by a pair of flexor/extensor muscles. These muscles received rotational commands from 192 output cells of the M1 model, while the M1 model received input from muscle-specific groups of sensory cells, each of which were tuned to fire over a range of muscle lengths. The M1 model had 384 excitatory and 192 inhibitory event-based integrate-and-fire neurons, with AMPA/NMDA and GABA synapses. Excitatory and inhibitory units were interconnected probabilistically. Plasticity was enabled in the feedforward connections between input and output excitatory units. Poisson noise was added to the output units for driving stochastic movements. The reinforcement learning (RL) algorithm used eligibility traces for synaptic credit/blame assignment, and a global signal (+1=reward, -1=punishment) corresponding to dopaminergic bursting/dipping. Eligibility traces were spike-timing-dependent, with pre-before-post spiking required. Reward (punishment) was delivered when the distance between the hand and target decreased (increased) [1].

RL learning occurred over 100 training sessions with the arm starting at 15 different initial positions. Each sub-session consisted of 15 s of RL training from a specific starting position. After training, the network was tested for its ability to reach the arm to target from each starting position, over the course of a 15 s trial. Compared to the naive network, the network post-training was able to reach the target from all starting positions. This was most clearly pronounced when the arm started at a large distance from the target. After reaching the target, the hand tended to oscillate around the target. Learning was most effective when recurrent connectivity in the output units was turned off or at low levels. Best overall performance was achieved with no recurrent connectivity and moderate maximal weights. Although learning typically increased average synaptic weight gains in the input-to-output M1 connections, there were frequent reductions in weights as well. Our model predicts that optimal motor performance is sensitive to perturbations in both strength and density of recurrent connectivity within motor cortex and that therefore the wiring of recurrent connectivity during development might be carefully regulated.

## References

[B1] Chadderdon GL Neymotin SA Kerr CC Francis JT Lytton WW Dopamine-based reinforcement learning of virtual arm reaching task in a spiking model of motor cortex International Conference on Cognitive and Neural Systems 16 Boston, MA

